# Rigidez Aórtica e Resposta aos Inibidores da Fosfodiesterase-5 em Pacientes em Tratamento para Disfunção Erétil: Papel Preditivo ou Epifenômeno?

**DOI:** 10.36660/abc.20240092

**Published:** 2024-04-04

**Authors:** Eduardo Tibiriça

**Affiliations:** 1 Instituto Nacional de Cardiologia - Coordenação de Ensino e Pesquisa Rio de Janeiro RJ Brasil Instituto Nacional de Cardiologia - Coordenação de Ensino e Pesquisa, Rio de Janeiro, RJ – Brasil

**Keywords:** Rigidez Vascular, Disfunção Erétil, Inibidores da Fosfodiesterase 5

A disfunção erétil (DE), um problema de saúde com patogênese multifatorial e alta prevalência, está diretamente correlacionada com a idade do paciente e está relacionada à disfunção endotelial e a um risco aumentado de doença cardiovascular (DCV).^1^ A disfunção endotelial vascular atualmente é considerada ser a ligação entre DE vasculogênica e doenças cardiometabólicas, incluindo hipertensão, diabetes e aterosclerose.^1 , 2^ A associação entre DE e DCV é reconhecida há muito tempo, e estudos sugerem que a DE pode ser um marcador independente de risco de DCV, incluindo a presença de doença arterial coronariana subclínica em homens assintomáticos.^3^

Nesse contexto, o uso generalizado de inibidores da fosfodiesterase tipo 5 (iPDE-5) tem levado mais pacientes com DE a procurar ajuda médica, proporcionando uma excelente oportunidade para a identificação e correção de possíveis fatores de risco cardiovascular.^4^ Além de problemas vasculares funcionais e estruturais acredita-se que alterações na circulação sistêmica que ocorrem durante a DE, distúrbios microcirculatórios locais e fibrose contribuam para a patogênese da DE.^5 , 6^ Além disso, foi demonstrado que a função microvascular dependente do endotélio peniano melhora após o uso contínuo de sildenafil em pacientes hipertensos com DE.^7^

Nesta edição da revista, Çiçek et al.^8^ descreveram um estudo clinicamente relevante que foi conduzido para investigar o suposto valor preditivo da rigidez aórtica na avaliação das respostas clínicas ao tratamento com iPDE-5 em pacientes com DE. A ecocardiografia transtorácica é um método não invasivo que pode ser usado para avaliar a variabilidade pulsátil, tensão e distensibilidade na aorta. Na verdade, a rigidez arterial pode ser facilmente avaliada calculando-se os parâmetros de elasticidade aórtica com a ecocardiografia transtorácica, que é um método de fácil aplicação e tempo eficiente, que não requer nenhum outro equipamento ou software.^9 , 10^ No entanto, alguns aspectos do trabalho de Çiçek merecem uma discussão mais aprofundada. Primeiro, os pacientes incluídos no estudo eram bastante jovens, com uma idade média de aproximadamente 45-50 anos. Notavelmente, a DE é cada vez mais prevalente com a idade: aproximadamente 40% dos homens são afetados aos 40 anos de idade, e quase 70% dos homens são afetados aos 70 anos;^11^ além disso, a idade é o parâmetro mais fortemente associado à DE.^11^ Além disso, embora não tenha havido diferença em relação à idade entre os três grupos de pacientes quando analisados em conjunto com ANOVA, uma comparação separada da média de idade e desvio padrão (DP) (usando testes t de Student) entre os grupos com DE grave e leve a moderada mostraram uma diferença significativa entre os dois grupos (p=0,0367), sugerindo que os pacientes com DE leve a moderada eram mais jovens do que aqueles com DE grave. Neste caso, a idade poderia ser considerada uma variável de confusão que poderia resultar na super ou subestimação do impacto da variável independente sobre a variável dependente. Esta desvantagem poderia ter sido resolvida pela inclusão de possíveis confundidores como variáveis de controle nos modelos de regressão; desta forma, teria sido possível controlar o impacto da variável de confusão. Qualquer efeito que a potencial variável de confusão tenha sobre a variável dependente seria observado nos resultados da regressão e permitiria a separação do impacto da variável independente.

Finalmente, na seção de Limitações do estudo, os autores reconheceram que seriam necessários estudos multicêntricos com séries maiores de pacientes para confirmar os resultados do presente estudo.

No entanto, os resultados de Çiçek et al^8^ sugerem que a deformação aórtica e a distensibilidade aórtica medidas de forma não invasiva usando ecocardiografia transtorácica poderiam ser marcadores adicionais para prever as respostas do paciente ao tratamento para DE com iPDE-5. Assim, estudos adicionais incluindo séries maiores de pacientes e análises estatísticas robustas são necessários.

## References

[1] De Leonardis F, Colalillo G, Agrò EF, Miano R, Fuschi A, Asimakopoulos AD (2022). Endothelial Dysfunction, Erectile Deficit and Cardiovascular Disease: An Overview of the Pathogenetic Links. Biomedicines.

[2] Konstantinovsky A, Tamir S, Katz G, Tzischinsky O, Kuchersky N, Blum N (2019). Erectile Dysfunction, Sleep Disorders, and Endothelial Function. Isr Med Assoc J.

[3] Miner M, Parish SJ, Billups KL, Paulos M, Sigman M, Blaha MJ (2019). Erectile Dysfunction and Subclinical Cardiovascular Disease. Sex Med Rev.

[4] Kloner RA, Jarow JP (1999). Erectile Dysfunction and Sildenafil Citrate and Cardiologists. Am J Cardiol.

[5] Belcaro G, Cesarone MR, Nicolaides AN, Vale J, Glass J, Lennox A (2000). Microcirculatory Studies in Erectile Disorders. Curr Med Res Opin.

[6] Verri V, Brandão A, Tibirica E (2015). The Evaluation of Penile Microvascular Endothelial Function Using Laser Speckle Contrast Imaging in Healthy Volunteers. Microvasc Res.

[7] Verri V, Nascimento AR, Brandao AA, Tibirica E (2021). Effects of Chronic Type 5 Phosphodiesterase Inhibition on Penile Microvascular Reactivity in Hypertensive Patients with Erectile Dysfunction: A Randomized Crossover Placebo-controlled Trial. J Hum Hypertens.

[8] Çiçek Ömer Faruk (2024). 1 Halil Ferat Öncel,2 Remzi Salar. Role of Aortic Stiffness in Predicting Response to Phosphodiesterase-5 Inhibitors in the Treatment of Erectile Dysfunction. Arq Bras Cardiol.

[9] Vitarelli A, Giordano M, Germanò G, Pergolini M, Cicconetti P, Tomei F (2010). Assessment of Ascending Aorta Wall Stiffness in Hypertensive Patients by Tissue Doppler Imaging and Strain Doppler Echocardiography. Heart.

[10] Stefanadis C, Stratos C, Boudoulas H, Kourouklis C, Toutouzas P (1990). Distensibility of the Ascending Aorta: Comparison of Invasive and Non-Invasive Techniques in Healthy Men and in Men with Coronary Artery Disease. Eur Heart J.

[11] Feldman HA, Goldstein I, Hatzichristou DG, Krane RJ, McKinlay JB (1994). Impotence and its Medical and Psychosocial Correlates: Results of the Massachusetts Male Aging Study. J Urol.

