# Supplementary material for: Rigidez Aórtica e Resposta aos Inibidores da Fosfodiesterase-5 em Pacientes em Tratamento para Disfunção Erétil: Papel Preditivo ou Epifenômeno?
Source: Arq Bras Cardiol. 2024 Apr 4;121(3):e20240092. [Article in Portuguese] doi: 10.36660/abc.20240092 (PMC11081094; doi:10.36660/abc.20240092)
Supplement: Translation

# Translation: Aortic Stiffness and the Response to Phosphodiesterase-5 Inhibitors in Patients Receiving Treatment for Erectile Dysfunction: Predictive Role or an Epiphenomenon?

**DOI:** 10.36660/abc.20240092i

**Authors:** Eduardo Tibiriça

**Affiliations:** 1 Rio de Janeiro RJ Brazil Instituto Nacional de Cardiologia - Coordenação de Ensino e Pesquisa, Rio de Janeiro, RJ – Brazil

**Keywords:** Vascular Stiffness, Erectile Dysfunction, Phosphodiesterase 5 Inhibitors

Erectile dysfunction (ED), a health problem with multifactorial pathogenesis and high prevalence, is directly correlated with patient age and is related to endothelial dysfunction and an increased risk of cardiovascular disease (CVD).^1^Vascular endothelial dysfunction is currently considered to be the link between vasculogenic ED and cardiometabolic diseases, including hypertension, diabetes, and atherosclerosis.^1 , 2^The association between ED and CVD has long been recognized, and studies suggest that ED may be an independent marker of CVD risk, including the presence of subclinical coronary artery disease in otherwise asymptomatic men.^3^

In this context, the general use of type 5 phosphodiesterase inhibitors (iPDE-5) has led more patients with ED to seek medical help, providing an excellent opportunity for the identification and correction of possible cardiovascular risk factors.^4^In addition to functional and structural vascular alterations in the systemic circulation that occur during ED, local microcirculatory disturbances and fibrosis are thought to contribute to the pathogenesis of ED.^5 , 6^Moreover, penile endothelium-dependent microvascular function has been shown to improve after continuous use of sildenafil in hypertensive patients with ED.^7^

In this issue of the journal, Çiçek et al.^8^described a clinically relevant study that was conducted to investigate the putative predictive value of aortic stiffness in the evaluation of clinical responses to treatment with iPDE-5 in patients with ED. Transthoracic echocardiography is a noninvasive method that can be used to assess pulsatile variability, strain, and distensibility in the aorta. In fact, arterial stiffness can easily be assessed by calculating aortic elasticity parameters with transthoracic echocardiography, which is an easy-to-apply and time-efficient method that does not require any other equipment or software.^9 , 10^Nevertheless, some aspects of Çiçek's work merit further discussion. First, the patients included in the study were quite young, with an average age of approximately 45-50 years. Notably, ED is increasingly prevalent with age: approximately 40% of men are affected at the age of 40 years, and nearly 70% of men are affected at the age of 70 years;^11^moreover, age is the parameter that is most strongly associated with ED.^11^Moreover, even though there was no difference concerning age among the three groups of patients when analyzed together with ANOVA, a separate comparison of the mean age and standard deviation (SD) (using Student's t-tests) between the groups with severe and mild-moderate ED showed a significant difference between the two groups (p=0.0367), suggesting that patients with mild-moderate ED were younger than those with severe ED. In this case, age could be considered a confounding variable that could result in the over- or underestimation of the impact of the independent variable on the dependent variable. This drawback could have been solved by the inclusion of possible confounders as control variables in the regression models; in this way, it would have been possible to control for the impact of the confounding variable. Any effect that the potential confounding variable has on the dependent variable would be observed in the results of the regression and allow for the separation of the impact of the independent variable.

Finally, in the Limitations section of the study, the authors acknowledged that multicenter studies with larger series of patients would be necessary to confirm the results of the present study.

Nevertheless, the results of Çiçek et al.^8^suggest that aortic strain and aortic distensibility measured noninvasively using transthoracic echocardiography could be additional markers for predicting patient responses to treatment for ED with iPDE-5. Thus, additional studies including larger series of patients and robust statistical analyses are warranted.
